# Effects of Power-Oriented Resistance Training With Heavy vs. Light Loads on Muscle-Tendon Function in Older Adults: A Study Protocol for a Randomized Controlled Trial

**DOI:** 10.3389/fphys.2021.635094

**Published:** 2021-02-18

**Authors:** Carlos Rodriguez-Lopez, Julian Alcazar, Jose Losa-Reyna, Noelia Maria Martin-Espinosa, Ivan Baltasar-Fernandez, Ignacio Ara, Robert Csapo, Luis M. Alegre

**Affiliations:** ^1^GENUD Toledo Research Group, Universidad de Castilla-La Mancha, Toledo, Spain; ^2^CIBER of Frailty and Healthy Aging (CIBERFES), Madrid, Spain; ^3^Department of Geriatrics, Hospital Virgen del Valle, Complejo Hospitalario de Toledo, Toledo, Spain; ^4^Faculty of Physiotherapy and Nursing, Universidad de Castilla-La Mancha, Toledo, Spain; ^5^Research Unit for Orthopaedic Sports Medicine and Injury Prevention, ISAG, University for Health Sciences, Medical Informatics and Technology, Hall in Tirol, Austria

**Keywords:** aging, physical function, strength training, power training, force-velocity, intensity

## Abstract

**Background:**

Power-oriented resistance training (PRT) is one of the most effective exercise programs to counteract neuromuscular and physical function age-related declines. However, the optimal load that maximizes these outcomes or the load-specific adaptations induced on muscle power determinants remain to be better understood. Furthermore, to investigate whether these adaptations are potentially transferred to an untrained limb (i.e., cross-education phenomenon) could be especially relevant during limb-immobilization frequently observed in older people (e.g., after hip fracture).

**Methods:**

At least 30 well-functioning older participants (>65 years) will participate in a within-person randomized controlled trial. After an 8-week control period, the effects of two 12-week PRT programs using light vs. heavy loads will be compared using an unilateral exercise model through three study arms (light-load PRT vs. non-exercise; heavy-load PRT vs. non-exercise; and light- vs. heavy- load PRT). Muscle-tendon function, muscle excitation and morphology and physical function will be evaluated to analyze the load-specific effects of PRT in older people. Additionally, the effects of PRT will be examined on a non-exercised contralateral limb.

**Discussion:**

Tailored exercise programs are largely demanded given their potentially greater efficiency preventing age-related negative consequences, especially during limb-immobilization. This trial will provide evidence supporting the use of light- or heavy-load PRT on older adults depending on individual needs, improving decision making and exercise program efficacy.

**Clinical Trial Registration:**

NCT03724461 registration data: October 30, 2018.

## Introduction

Resistance training has become a core component of exercise prescribed for older adults due to its capacity to mitigate the effects of aging on neuromuscular function and improve functional capacity (Liu and Latham, 2009; Peterson et al., 2010; Borde et al., 2015; Lopez et al., 2018). Recent recommendations for resistance training for older adults suggest to include power-oriented exercises (i.e., concentric movements performed as fast as possible) (Fragala et al., 2019). This is because muscle power is more closely related to physical function than muscle strength (Bean et al., 2003; Alcazar et al., 2020) and, therefore, considered the most robust biomarker of age-related neuromuscular decline. Moreover, power-oriented resistance training (PRT) has shown greater effects on physical function than traditional resistance training in older adults (Tschopp et al., 2011; Byrne et al., 2016; Ramirez-Campillo et al., 2016).

Despite its undisputed functional relevance, scant data are available to inform the proper prescription of PRT in older adults (Steib et al., 2010; Borde et al., 2015; Csapo and Alegre, 2016). The general goal of PRT is to execute concentric muscle actions with maximal velocity to maximize power output. However, a large variety of exercises (Franchi et al., 2019; Van Roie et al., 2020), resistance types (i.e., pneumatic, isotonic, body weight) (Sayers and Gibson, 2010; Van Roie et al., 2020), and intensities (i.e., loads used) (de Vos et al., 2005; Reid et al., 2015) can be found in the literature. In the last years, several previous studies have been performed with the intent to determine the optimal load for power development in older people (de Vos et al., 2005; Reid et al., 2015; Richardson et al., 2019). Summarizing these works in a systematic review, Katsoulis et al. (2019) concluded that a wide range of intensities may be used to improve muscle power and physical function in older adults. However, the variability of outcome measures and limited power of the studies included prevented the authors from performing a meta-analysis. For this reason, the authors recommended that further investigations comparing different intensities or frequencies of training and addressing force- or velocity-dependent components of the power adaptations should be conducted (Katsoulis et al., 2019). Therefore, well-designed studies using standardized testing procedures to compare the effects of PRT as performed using different loads in older adults are warranted.

To improve the quality of evidence, future studies should (i) compare volume × load-matched interventions (Csapo and Alegre, 2016); (ii) include a control period before interventions in order to account for the error of measurement, biological variability and training effect during testing (Hopkins, 2000); and (iii) use unilateral exercise model designs to allow for within-person comparisons to be made (Burd et al., 2010; Mitchell et al., 2012; Alegre et al., 2015; Pandis et al., 2017). This latter recommendation helps to increase statistical power by reducing between-person variability and limiting co-founding factors like nutrition, sleep, and physical activity (MacInnis et al., 2017). Additionally, unilateral exercise models provide the opportunity to investigate the cross-education phenomenon, which may help to estimate the neural and muscular contributions to PRT-related strength gains. Cross-education is mainly characterized by an increase in maximal voluntary force observed in the contralateral untrained limb (Munn et al., 2004; Lee and Carroll, 2007). Previous evidence was focused on the adaptations provoked by traditional heavy resistance training on maximal voluntary force (i.e., 1RM and maximal isometric force), whereas limited evidenced is available about cross-education phenomenon during isometric (Hester et al., 2019a) or dynamic (Hester et al., 2019b) ballistic actions following PRT in older people. Moreover, it could be expected that the load used during PRT influences the magnitude of the cross-education phenomenon (Cirer-Sastre et al., 2017; Colomer-Poveda et al., 2019, 2020). Hence, it deserves to be examined whether specific cross-education adaptations might be driven by different loads during PRT. This would enhance training prescription during limb immobilization and rehabilitation programs, especially in older people who are susceptible to long-term immobilizations after falling (e.g., hip fracture) associated to substantial neuromuscular declines and attenuated retraining capacity (Suetta et al., 2009).

Considering the above suggestions, this manuscript serves to present a protocol for a series of studies comparing the effects of heavy- vs. light-load PRT in older people. The main aim of this project is to assess the adaptations provoked by two volume × load-matched PRT programs using heavy vs. light loads in older adults (goal 1). The secondary aim is to investigate the adaptations provoked by heavy- vs. light-load PRT on the contralateral limb in older adults (goal 2).

## Methods/Design

### Experimental Approach

**FIGURE 1:**
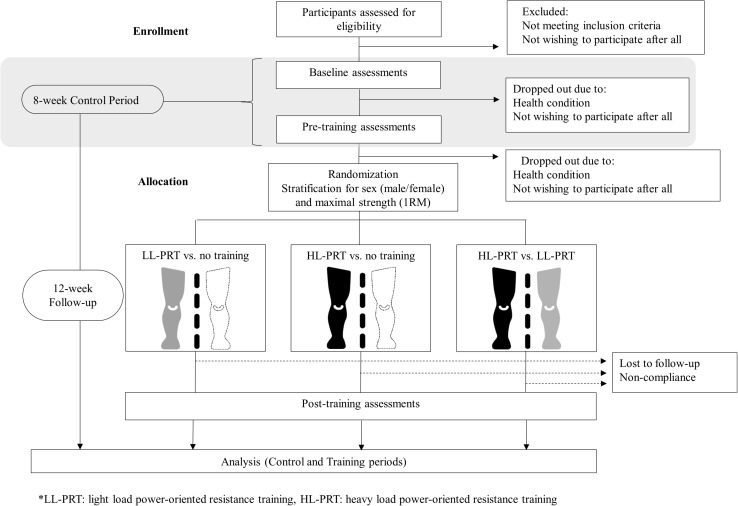
Flowchart of the study protocol.

The present project and study protocol were registered in clinicaltrials.gov (ID: NCT03724461, October 30th, 2018) and approved by the Clinical Research Ethics Committee of the Complejo Hospitalario de Toledo (Spain). The study protocol will be conducted following the SPIRIT (Standard Protocol Items: Recommendations for Interventional Trials) statement (Chan et al., 2013). The intervention and data collection will be conducted at the University of Castilla-La Mancha, Toledo (Spain). This is a within-person randomized trial, including an 8-week control period followed by 12 weeks of PRT (Figure 1). The mean changes observed during the 8-week control period will be quantified to determine the possible extent of spontaneous changes in all outcome measures. After completing the control period, the participants will be randomized to one of the following study arms based on the treatment applied to their lower-limbs during the 12-week PRT program: (i) one leg will perform a light-load PRT and the other leg will not perform any exercise; (ii) one leg will perform a heavy-load PRT and the other leg will not perform any exercise; and (iii) one leg will perform a heavy-load PRT and the other leg will perform a light-load PRT. Assessments will be conducted at baseline (week 0), at the end of the control period (week 8), and following the 12-week PRT (week 20).

### Participants

The sample size was determined by an *a priori* power analysis using G^∗^Power v3 software for MS Windows^®^ (Faul et al., 2007). The effect size value used for calculations was *d* = 0.25 based on a previous study comparing peak power adaptations after a 12-week heavy- vs. light-load PRT in older adults (de Vos et al., 2005). Then, we considered three levels for the between-subject factor (i.e., one for each study arm) and three levels for the within-subject factor (i.e., three repeated measures) setting the alpha error probability at 0.05, a 1−β of 0.95 and assuming a correlation among repeated measures of *r* = 0.8. The model indicated a minimum total sample size of 24 individuals, but considering a 25% dropout rate, we have decided to enroll at least 30 participants in total. People aged 65 years and over, non-institutionalized and without serious mobility limitations will be encouraged to participate. Principally, the volunteers will be recruited among participants involved in non-exercise studies previously conducted by our research group, local advertisements posted in public places and informative talks about healthy aging. After accepting to participate, the volunteers will undergo a physical examination by a geriatrician. The criteria for exclusion are: (i) frailty status (Fried et al., 2001) or low levels of physical function (i.e., Short Physical Performance Battery <7 points) (Guralnik et al., 1994), (ii) neurological, musculoskeletal, or other disorders that might preclude subjects from completing the resistance training and all performance tests, (iii) uncontrolled hypertension, unstable or exercise-induced angina pectoris or myocardial ischemia or any other medical condition that would interfere with testing or increase subjects’ risk of complications during exercise, (iv) history of regular resistance exercise during the previous 3 years, and (v) total or partial knee replacement (i.e., prosthesis). All participants will be informed orally and in written form about the purpose, procedures, benefits, risks, and potential discomfort related to study participation before signing a consent form. This study will be conducted in accordance with the Helsinki declaration.

### Randomization and Allocation of Participants

After completing the control period, the participants will be stratified by their sex and 1RM values and randomly allocated in equal numbers to the three intervention groups. Then, the treatment applied to the subjects’ legs will be determined by coin toss.

### Interventions

All participants will complete a 12-week PRT programs targeting the lower limbs twice a week (total of 24 sessions). Training sessions will be performed on a horizontal leg press device that includes a weight stack system (Selection MD, Technogym, Italy) where participants will execute unilateral repetitions. For standardization, participants will be requested to fully extend their leg from a common starting position (knee and hip joint angles of 90° and 70°, respectively; full extension = 180°). Each exercise session will start with a 5-min warm-up on a cycle-ergometer at a low self-regulated intensity and a crank velocity of approximately 70 rpm. Then, the lower limbs to be trained will undergo a specific warm-up consisting of one set of 10 repetitions at 50% of the individual one repetition maximum (1-RM), with repetitions executed at submaximal velocity. The PRT performed with light loads (LL-PRT) will consist of six sets of 12 repetitions using a load equivalent to 40% 1-RM, whereas heavy-load PRT (HL-PRT) will comprise six sets of six repetitions with a load equivalent to 80% 1-RM. Therefore, the total volume × load will be matched between the training programs to minimize bias related to unequal training volumes. In addition, the 1-RM will be re-assessed in 4-week intervals to adjust training loads and ensure progressive overload. For both training programs, the participants will be instructed and verbally encouraged to perform the concentric phase of each repetition as fast and strong as possible (i.e., ballistic approach), whereas during the eccentric phase the load will be lowered in a controlled manner in 3 s and 2 s for LL-PRT and HL-PRT, respectively. These unequal durations serve to balance the greater volitional effort required to retain a heavier load compared with a light load during the eccentric phase (Rodriguez-Lopez et al., 2020). Sets will be interspersed by 2-min rest periods. In subjects in whom both lower limbs will be trained (i.e., HL-PRT vs. LL-PRT group), an additional rest period of 1-min will be included between each leg, and the leg trained first will alternate between sessions. To warrant adequate recovery, all training and testing sessions will be separated by a minimum of 48 h. Participants will be required to attend to at least 80% of sessions. Those unable to participate for 10 days or more will be excluded from the study. Furthermore, subjects will be asked to maintain their regular physical activity levels and diet, and to inform study administrators about any changes in their medications. All training sessions and tests will be attended individually, supervised by an experienced sport scientist, and conducted approximately at the same time every day.

### Outcomes

All tests to be performed at each measurement point will be completed on three separate days. In the morning of the first day, blood samples, anthropometric, and body composition data will be collected with the participants in a fasting state. After a light and standardized breakfast, participants will complete a physical function test battery. On the second day, muscle size and architecture of the mid-thigh along with patellar tendon mechanical properties will be assessed. On the third day, the unilateral neuromuscular performance of the lower limbs will be assessed through isometric and dynamic tests. Additionally, a progressively loaded five-repetition sit-to-stand (STS) test will be conducted. Before the baseline assessments, at least two familiarization sessions will be completed to instruct participants in the proper technique of the neuromuscular tests. A detailed scheme of the assessments and outcomes that will be evaluated during this study is shown in Table 1.

**TABLE 1:** Overview of the outcomes, time points of measurements, and treatments.

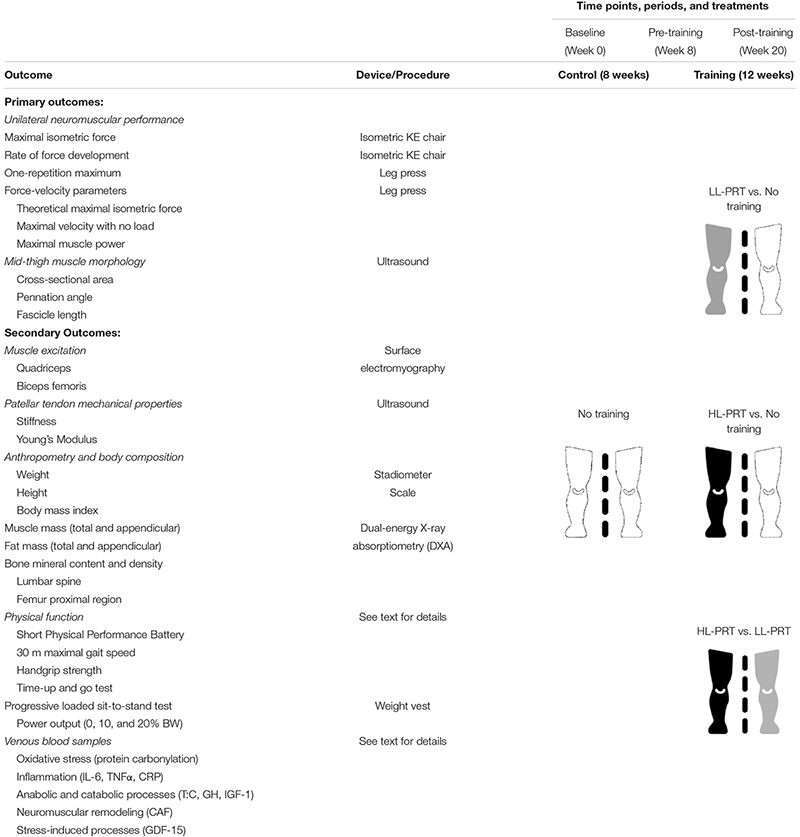

*KE, knee extension; BW, body weight; HL–PRT, heavy load power–oriented resistance training; LL–PRT, light load power–oriented resistance training; IL–6, Interleukin 6; TNFα, tumoral necrosis factor alpha; CRP, C-reactive protein; T:C, testosterone:cortisol ratio; GH, growth hormone; IGF–1, insulin growth factor-1; CAF, C–terminal agrin fragment; GDF–15, growth differentiation factor 15.*

### Primary Outcomes

#### Maximal Isometric Force (MIF) and Rate of Force Development (RFD) of the Knee Extensors

For isometric strength tests, participants will be seated on a custom-built isometric chair (Telju Fitness, Spain) with knee and hip angles of 90° and 120°, respectively (180° full extension). An ankle brace together with a steel cable with minimal compliance will be used to connect the lower leg to a strain gauge cell (Linear Force SmartLead, Noraxon, United States) to register knee extension force at 1,500 Hz. Then, participants will be instructed to perform several maximal voluntary isometric contractions (MVIC), extending their knee as fast and strong as possible and holding the contraction for 3 s after the cue “ready, set, go!.” Strong verbal encouragement and visual feedback will be given in each attempt to ensure maximal efforts. Five adequate trials (separated by 60 s) will be acquired. Then, the MIF will be determined as the highest force value registered, and the RFD will be calculated as the linear slope of the time-force curve between 0–50, 0–100 and 0–200, 0–400 ms and at the maximal point of the time-RFD curve (RFD_max_).

#### Force-Velocity Relationship and Maximal Dynamic Strength in the Unilateral Leg Press Exercise

The assessments will be conducted on a horizontal leg press device (Selection MD, Technogym, Italy) instrumented with a force plate (Type 9286BA, Kistler, Switzerland) and a linear position transducer (Linear encoder, Chronojump Boscosystem, Spain). A detailed description and validation of the systematic procedure that will be followed to obtain the force-velocity relationship and maximal dynamic strength in older adults has been previously published (Alcazar et al., 2017). Briefly, the participants will perform two sets of two repetitions with the starting load equivalent to 40% of their body mass being gradually increased (5–20 kg increments) until the 1-RM is reached (failure was defined as not being able to lift a load within two attempts). From the starting position (knee and hip joint angles of 90° and 70°, respectively; full extension = 180°), the repetitions will be executed as fast as possible during the concentric phase, whereas self-preferred eccentric velocity will be demanded to warrant maximal performance in the subsequent repetition. The processing of force and velocity signals has been described previously (Rodriguez-Lopez et al., 2020). Shortly, force and velocity values from the most powerful attempts of each load (i.e., highest mean power value) will be fitted in a linear model, distinguishing between first and second repetitions (i.e., concentric-only vs. eccentric-concentric performance). Then, the theoretical maximal isometric force and maximal velocity with no load will be estimated through extrapolation of the linear regression line (force- and velocity intercepts, respectively). In addition, the maximal muscle power as well as the force and velocity at which it is produced will be determined (Alcazar et al., 2017).

#### Mid-Thigh Muscle Size and Architecture

The participants will lie on an examination table in supine position and with the legs slightly flexed with the aid of a foam roller placed beneath the knee. To allow for fluid shifts to occur, this position will be held for 15 min before examination. Brightness mode ultrasound (MyLab 25, Esaote Biomedica, Genova, Italy) images will be taken with a 50 mm, 10–15 MHz linear-array probe. The scans will be acquired at 50% of the distance between the greater trochanter and the inferior border of the lateral condyle of the femur. For muscle size quantification, three transversal panoramic scans of the rectus femoris and the vastus lateralis muscles will be obtained using the extended field of view image stitching technique. For this purpose, the examiner will acquire images by moving the prove from the medial aponeurosis of the rectus femoris to the lateral border of the vastus lateralis at constant velocity. Then, three longitudinal images of the vastus lateralis will be obtained with the probe positioned at an individually determined optimal location (characterized by parallel aponeuroses and consistency of the fascicle orientation) and aligned with the fascicle plane. Abundant transmission gel will be applied to ensure acoustic coupling with minimal pressure to avoid muscle deformation. All scans will be subsequently analyzed using Fiji image analysis software (Schindelin et al., 2012). For measurements of muscle size of the rectus femoris and vastus lateralis, the muscles’ aponeuroses will be traced to quantify the cross-sectional area. The vastus lateralis muscle architecture will be analyzed through the Simple Muscle Architecture Analysis tool for Fiji (Seynnes and Cronin, 2020). Basically, this is an automated analysis process that highlights aponeuroses and fascicles to obtain the dominant fascicle orientation (i.e., pennation angle) and estimate fascicle length.

### Secondary Outcomes

#### Quadriceps and Biceps Femoris Muscle Excitation

Wireless surface electromyography (DTS EMG sensors and Desktop DTS, Noraxon United States) will be employed to acquire the excitation levels of the quadriceps femoris muscle (rectus femoris, vastus medialis, and vastus lateralis) and the long head of the biceps femoris muscle during the unilateral knee extension and leg press neuromuscular performance tests and the assessment of the patellar tendon mechanical properties (see below). The SENIAM recommendations will be followed when placing bipolar electrodes (HEX Dual Electrodes, Noraxon, United States) onto each muscle belly after proper skin preparation (Hermens et al., 2000). Raw surface electromyography signals will be captured at 1,500 Hz, amplified and filtered with a band-pass filter between 10 and 500 Hz (common mode rejection ratio >100 dB, input impedance >100 MΩ, and gain = 500) before any other signal processing. Unless otherwise stated, raw surface electromyography signals will be rectified and smoothed by calculating the root mean square with a 100 ms time window. The resultant signals amplitudes will be normalized to the muscle-specific maximum values measured during knee extension and flexion MVIC testing (EMG AMP,% MVIC). Furthermore, total cumulative muscle excitation will be calculated as the integrated area under the EMG AMP curve (%MVIC × s). Electromyographic and force signals will be synchronously processed within commercial software (MyoResearch 3.10, Noraxon, United States).

#### Patellar Tendon Mechanical Properties

The morphology of the patellar tendon at rest will be assessed with the same ultrasound system also used for measurements of muscle size and architecture. For this purpose, the participants will be accommodated (after a 5-min cycle ergometer warm-up) on the custom-built chair used for isometric strength tests, with knee and hip angles of 90° and 120°, respectively (180° full extension), and their lower leg connected to a strain gauge cell (see above). The extended field of view mode will be used to acquire images of the resting patellar tendon in the sagittal plane to determine its length as the distance between the tibial tuberosity and the patellar apex. In addition, the cross-sectional area of the patellar tendon will be measured at 25, 50, and 75% of the tendon length.

**FIGURE 2:**
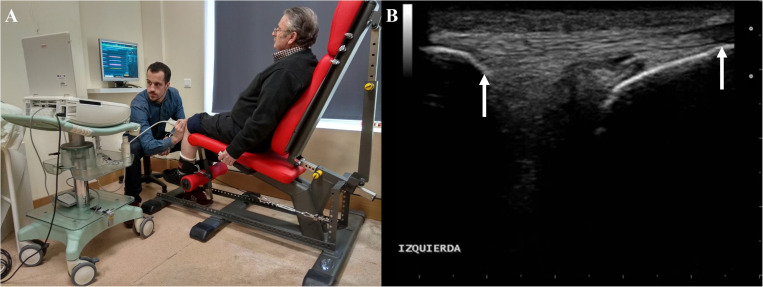
Demonstrative example of the experimental setting for acquisition of *in vivo* patellar tendon behavior during isometric ramp contractions **(A)**. Field of view of ultrasound images collected from the patellar tendon **(B)**. White arrows indicate points on patella **(left)** and tibial tuberosity **(right)** that will be tracked to determine tendon elongation.

Prior to measurements of tendon mechanical properties, participants will perform several submaximal isometric ramp contractions with constant loading and visual feedback for tendon conditioning. Afterward, the participants will first execute three MVIC of the knee extensors, followed by three further maximal contractions of the knee flexors, with all contractions being separated by 60-s rest intervals. These tests will serve for the normalization of the electromyographic signals and to establish the loading rate for the ramp isometric contractions in which tendon elongation will be measured. Then, the ultrasound probe will be positioned on the patellar tendon in the sagittal plane, with both the patella and tibial tuberosity in the field of view. A demonstrative image of this experimental setting is shown in Figure 2. Ultrasound video sequences will be recorded at 24 Hz while participants perform eight appropriate ramp contractions of the knee extensors. The loading rate will be standardized, such that participants gradually increase the level of force produced from 0 to 80% of maximal isometric force over 4 s and then relax at the same controlled rate. The acquired force signals superimposed over a target template will be shown on a screen to provide instant feedback to participants (MyoResearch XP, Noraxon, United States). One minute of rest will be granted between trials.

All image processing will be performed offline. First, tendon elongation will be measured as the proximodistal component of the displacement of the patellar apex relative to the tibial plateau. The points of interest will be tracked using a semi-automated software (Tracker 4.91)^1^, allowing to obtain the continuous force-tendon length data for the subsequent analysis of tendon mechanical properties. Force and electromyographic signals (1,500 Hz) will be synchronized with ultrasound video (24 Hz) data using an MS Excel^®^ spreadsheet. The further processing will be performed using custom-made MATLAB^®^ routines (MATLAB R2014b, MathWorks, Natick, MA, United States). The knee extension torque will be calculated as the product of knee extension force and the distance between the center of rotation of the knee and the position of the ankle brace just proximal to the malleoli. Then, this product will be divided by the individual patellar tendon moment arm, estimated from femoral length (Visser et al., 1990), to obtain tendon force. Tendon stress will be calculated by dividing tendon force by the average of the patellar tendon cross-sectional area as measured at 25, 50, and 75% of tendon length. Tendon strain will be computed as the change in length in relation to resting tendon length. Individual force-tendon elongation curves will be fitted with second or third order polynomials. Then, tendon stiffness (Δforce/Δelongation) and Young’s modulus (Δstress/Δstrain) will be calculated as the slope of the fitted polynomials in the final 10% of force-elongation and stress-strain curves, respectively, of the weakest valid trial.

#### Anthropometric and Body Composition Assessments

Participants height, body mass, and body mass index will be assessed using a stadiometer and scale device (Seca 711, Seca, Hamburg, Germany). Total and appendicular body composition (i.e., absolute and relative bone, muscle and fat content) will be determined using a calibrated dual-energy X-ray absorptiometry (DXA) device (Hologic Series Discovery QDR densitometer; Hologic, Bedford, MA, United States). In addition to the whole body scans, bone mineral content and density will be determined from the lumbar spine (L1–L4) and the proximal region of both femurs (total hip, greater trochanter, intertrochanter, Ward’s triangle, and femoral neck). All DXA scans will be analyzed using Physician’s Viewer, APEX System Software Version 3.1.2 (Hologic, Bedford, MA, United States).

#### Physical Function Test Battery

A wide battery of test will be used to evaluate the physical function of participants. Firstly, the Short Physical Performance Battery will be conducted, which evaluates static balance, 4-m habitual gait speed and five-repetition sit-to-stand time (Guralnik et al., 1994). In addition, 4.5-m habitual gait speed and 30-m maximal gait speed will be also examined. The handgrip strength will be registered during a maximal isometric contraction using a digital dynamometer (Takei TKK 5401, Tokyo, Japan). For this purpose, participants will be instructed to let their arms hang straight and slightly abduct their shoulders. Finally, participants will complete a 3-m timed up and go test, which consists of getting up from a chair, walking around a cone located 3-m in front of the chair and sitting down again as fast as possible. Two appropriate trials will be collected from each test, registering the better of both performances.

#### Progressively Loaded 5-Repetition Sit-to-Stand Test

In addition to the tests included in the Short Physical Performance Battery, participants will perform an additional repeated sit-to-stand test with increasing loads. Starting from a seated position on a standardized armless chair with their feet over a force-plate and their arms crossed over the chest, they will be encouraged to complete five repetitions (rising to a full standing position and return to the original sitting position) as rapidly as possible. The test will be conducted under unloaded and loaded conditions: 0%, 10%, and 20% of the participant’s body mass. The better of two adequate trials in each loading condition will be registered. One minute of passive recovery will be guaranteed between trials. A video camera (HD Pro Webcam C920 1080p, 30 Hz, Logitech, Switzerland) will record all trials from the sagittal plane. Concentric and eccentric phases of the movement will be detected offline examining force and video signals synchronized within a specialized software (MyoResearch 3.10, Noraxon, United States). Mean force will be collected with the force plate. STS vertical displacement will be calculated as leg length (from the superior border of the greater trochanter of the femur to the inferior border of the calcaneus bone) minus the height of the chair from the top of the force plate (0.43 m). Then, mean velocity of each STS repetition will be calculated as the ratio between STS vertical distance and time elapsed during the concentric phase. Finally, mean power during each repetition will be determined as the product of mean force and mean velocity values.

#### Blood Samples Collection

Blood samples will be collected at rest after an overnight fast (>12 h) from an antecubital vein in different tubes containing thixotropic gel, sodium heparin and ethylenediaminetetraacetic acid (EDTA) (BD Vacutainer, Stockholm, Sweden). Samples will be collected at least 72–96 h apart from any exercise or testing session. Samples collected in the sodium heparin and EDTA tubes will be immediately centrifuged (1200 *g*, 10 min and 4°C) whereas thixotropic gel tubes will be kept at room temperature (22–24°C) for 30 min before being identically centrifuged. Samples will be analyzed immediately for routine clinical chemistry measurements and then, they will be put into 500-μL aliquots and frozen at −80°C for later analysis. This further analysis will determine the circulation levels of biomarkers related with oxidative stress, inflammation, anabolic and catabolic processes, neuromuscular remodeling and stress-induced processes. More specifically, oxidative stress will be determined through immunoblot detection of protein carbonylation with commercially available kits, levels of interleukin-6, tumoral necrosis factor alpha (TNFα), and C-reactive protein will be assessed as inflammatory markers and the testosterone: cortisol ratio, growth hormone, insulin growth factor-1, and creatine kinase will be analyzed as biomarkers of anabolic and catabolic processes with a high-sensitivity magnetic multiplex assay (Bio-Plex Multiplex System, Bio-Rad, CA, United States). Furthermore, C-terminal agrin fragment (CAF), as a neuromuscular remodeling biomarker, and growth differentiation factor (GDF-15) as a biomarker for stress-induced processes will be determined using commercially available enzyme-linked immunosorbent assay (ELISA) kits according to the manufacturer’s protocol. All samples collected for a given individual will be assessed within the same sample plate.

### Harms

Interventions and procedures considered in this study are minimally invasive, with a low risk of adverse events. Participants may develop delayed onset muscle soreness during the first sessions, diminishing after few sessions due to the repeated bout effect (Byrne et al., 2006). Since the exercise will be directly supervised by an experienced sport scientist, the risk of injury is minimal. Potential acute or persistent exercise-related adverse events will be brought to the attention of a study physician and discussed with both the participant and the project leader before making joint, informed decisions concerning their treatment and the further participation in the study.

### Statistical Analysis

Data will preferentially be presented as mean ± standard deviation. Normality of distribution will be assessed by the Shapiro–Wilk test and log-transformed in case of non-normal distribution. As preference, the outcomes will be registered as the absolute (Post – Pre) and relative [(Post – Pre)/Pre] change during the 8-week control period and the 12-week PRT program. Given the hierarchical structure and correlated data contained in our investigation (each participant will contribute to observations in two different groups), the existence of partially paired data must be considered for comparison purposes. Thus, linear mixed effect models will be used to assess the goals proposed for the present investigation. To assess the adaptations provoked by two volume × load-matched PRT programs using heavy vs. light loads in older adults (goal 1), changes noted in the legs undergoing HL-PRT and LL-PRT (i.e., week 20-week 8) and those noted in the same legs during the control period (i.e., week 8-week 0) will be compared with treatment (HL-PRT vs. LL-PRT vs. control) as a fixed factor, participants as a random factor and baseline values as a covariate. To investigate the adaptations provoked by heavy- vs. light-load PRT on the contralateral limb in older adults (goal 2), changes noted in the contralateral non-exercising legs of those legs performing either HL- or LL-PRT (i.e., week 20-week 8) and those noted in the same legs during the control period (i.e., week 8-week 0) will be compared with treatment (HL-PRT vs. LL-PRT vs. control) as a fixed factor, participants as a random factor and baseline values as a covariate. The models will be calculated considering maximum likelihood estimation and the best-fitting covariance structure. Bonferroni corrections will be applied for the *post hoc* pairwise comparisons and the Cohen’s *d* effect sizes will be calculated and classified as trivial (0.20), small (0.20–0.49), moderate (0.50–0.79), and large (>0.8) (Cohen, 1988). Potential adverse events and drop-outs will be compared between groups by Chi-squared tests. All statistical analyses will be performed using IBM SPSS Statistics for Windows Version 24.0 (IBM Corp. Armonk, NY, United States), and the level of significance will be set at α = 0.05.

## Discussion

The main purpose of the current project is to increase the knowledge about the effect of training intensity of PRT in older adults. Exploring specific adaptations induced by HL-PRT and LL-PRT will provide valuable data informing the prescription of resistive training in this population. The interindividual variability of training responses found in older adults recommends that exercise interventions be tailored according to individual deficits (Ramírez-Vélez and Izquierdo, 2019). For example, an impaired muscle power output may result from a reduced ability to produce force at slow velocities (i.e., deficit with heavy loads), limitations to produce force at high velocities (i.e., deficit with light loads), or even a combination of both (Alcazar et al., 2018). Although both deficits have been associated with poor functional performance, low quality of life and frailty (Alcazar et al., 2018), it seems reasonable to expect that training success rates would be greater if the PRT prescribed accounted for individual needs. In this sense, previous studies comparing PRT using different loads have found force- (de Vos et al., 2005; Orr et al., 2006) or velocity-dependent (Englund et al., 2017) specific adaptations for heavier and lighter loads, respectively. Conversely, studies using mechanical work-matched approaches (Macaluso et al., 2003) or those studies conducted in mobility-limited older adults (Reid et al., 2015) have not confirmed these specific adaptations. Against this background, this project will consider the recommendations of Katsoulis et al. (2019) and expand our knowledge concerning the functional benefits of individually tailored PRT in older age. In addition, our study will allow for mechanistic insights into the specific neuromuscular adaptations induced by HL-PRT and LL-PRT. Despite impaired neuromuscular excitation seems to be an important limitation for power production in older adults (Klass et al., 2008; Clark et al., 2010), other mechanisms such as muscle morphology and muscle-tendon behavior play an important role. For example, improvements in muscle mass, fascicle length, and pennation angle (and consequently increased physiological cross-sectional area of the muscle) have been linked to an enhanced muscle power production after a 6-weeks plyometric training intervention in older adults (Franchi et al., 2019). Besides that, high-loading exercises like heavy resistance training or PRT have showed to induce changes in the muscle-tendon complex (Hoffrén et al., 2012; Eriksen et al., 2019), modeling tendon behavior as a force transducer during the former, and as an elastic energy storage and power amplifier during the latter (Magnusson and Kjaer, 2019). This might be especially relevant during actions involving the stretch-shortening cycle, which induces a potentiation mechanism that mitigate power production limitations seen during pure concentric actions in older adults (Navarro-Cruz et al., 2019). In conjunction, these muscle power determinants that will be addressed in this study could be closely related with an expected improvement of functional performance (Tschopp et al., 2011; Byrne et al., 2016). In this sense, we aimed to evaluate the protective role of PRT against disability analyzing functional performance under different tasks and load-capacity conditions that will be obtained from the progressive loaded sit-to-stand test. Parallel to these adaptations, PRT has proved efficacy reinforcing bone health of postmenopausal women (Stengel et al., 2005; Hamaguchi et al., 2017) and as a suitable exercise to improve metabolic profile and body composition of older individuals (Ihalainen et al., 2019).

Regarding the second aim of this study (i.e., cross-education phenomenon), we expect to observe this phenomenon as most of its determinants will be fulfilled. The magnitude of cross education is greater in untrained participants and after training with complex and high demanding tasks as could be PRT (Lee and Carroll, 2007; Cirer-Sastre et al., 2017; Colomer-Poveda et al., 2019). Furthermore, the cross-education has been previously observed in older people after ballistic training (Hester et al.,2019a,b). However, the impact of the load used during PRT in cross-education in older adults remains to be elucidated. In a previous study conducted in older adults comparing the mechanical characteristics of light- vs. heavy-load PRT (40 vs. 80% 1-RM) (Rodriguez-Lopez et al., 2020), the muscle excitation registered (i.e., EMG amplitude) was similar when both training programs were executed until the same degree of fatigue was reached during the set. Since muscle excitation could be considered a proxy of neural excitation (Vigotsky et al., 2018), a major determinant of the cross-education phenomenon, it could be hypothesized that small or no differences will be found in the magnitude of the cross-education effects yielded by HL-PRT and LL-PRT in our study. However, other factors such as the anabolic systemic response to resistance training may influence on cross-education-derived adaptations, and thus, some differences may arise between using heavy vs. light loads in PRT in older adults.

In conclusion, the results of this study will shed light about the influence of load magnitude on PRT neuromuscular adaptations in older adults. Furthermore, the original design of this study brings the opportunity to investigate possible cross-education adaptations on an untrained contralateral leg. Hence, this study will aid to design tailored training programs in older adults, even during limb-immobilization or for clinical populations where unilateral training is recommended.

## Data Availability Statement

The raw data supporting the conclusions of this article will be made available by the authors, without undue reservation.

## Ethics Statement

Written informed consent was obtained from the individual(s) for the publication of any potentially identifiable images or data included in this article.

## Author Contributions

CR-L, JA, JL-R, IB-F, and NM-E performed the material preparation, data collection, and analysis. CR-L wrote the first draft of the manuscript. All authors listed qualify for authorships due their substantial contributions to the work, contributed to the study conception and design, commented on previous versions of the manuscript, and read and approved the final version of the manuscript.

## Conflict of Interest

The authors declare that the research was conducted in the absence of any commercial or financial relationships that could be construed as a potential conflict of interest.

## Footnotes

1 http://physlets.org/tracker/
